# Barriers, facilitators, and implementation strategies for the initiation of Child Death Review system in Japan: a modified Delphi method study

**DOI:** 10.1186/s12913-022-08668-x

**Published:** 2022-12-05

**Authors:** Haruko Yatake, Ai Aoki, Atsushi Numaguchi, Kenji Takehara

**Affiliations:** 1 Department of Health Policy, National Center for Child Health and Development, 2-10-1 Okura, Setagaya-ku, Tokyo Japan; 2 Department of Emergency and Critical Care Medicine, Nagoya University Graduate School of Medicine, 65 Tsurumai-cho, Showa-ku, Nagoya, Aichi Japan

**Keywords:** Child death review, Barriers, Facilitators, Implementation strategies, Prevention, Child mortality, Japan

## Abstract

**Background:**

To further curb preventable child deaths, some countries have implemented Child Death Review (CDR). CDR is a comprehensive multidisciplinary process that investigates, reviews, and registers all child deaths to consider prevention strategies. This study deciphered the barriers, facilitators, and implementation strategies in Japan.

**Methods:**

This study used a three-round modified Delphi method. The expert panel consisted of local government officers and health professionals responsible for the CDR pilot project in Japan. As a modification, the initial list of barriers, facilitators, and implementation strategies to address each barrier and facilitator was prepared based on project reports and interviews with local government officers. Throughout the three rounds, the panel evaluated predefined barriers and facilitators, suggested and evaluated additional items, and appraised the potential effectiveness of the implementation strategies on barriers and facilitators which they were meant to address. The importance of barriers and facilitators, and the potential effectiveness of implementation strategies were evaluated using 5-point Likert scale. The priority of the combinations of barriers, facilitators, and implementation strategies were determined considering their importance and effectiveness.

**Results:**

A total of 31 experts participated in the panel. Response rates were 96.8%, 80.6%, and 90.3% for the first, second, and third rounds, respectively. A total of 13 barriers, eight facilitators, and 72 implementation strategies corresponding to the barriers and facilitators reached consensus. At the national government level, a barrier-strategy combination of “lack of legislation (barrier)” and “legislation for CDR (strategy),” and a facilitator-strategy combination of “good multi-agency collaboration (facilitator)” and “official notices from the national government (strategy)” were at the highest priority. At the local government level, combinations of “lack of legislation (barrier)” and “constant budget allocations (strategy),” “lack of legislation (barrier)” and “citizens’ acceptance (strategy),” and “good multi-agency collaboration (facilitator)” and “appointment of a full-time staff (strategy)” were at the highest priority.

**Conclusion:**

This study demonstrated that legislation is the key to better implementation of CDR in Japan. Legislation can address various barriers such as personal information collection, multi-agency collaboration, high workload, and budget instability. Without legislation, careful strategies must be taken to solve difficulties caused by its absence.

**Trial registrations:**

None.

**Supplementary Information:**

The online version contains supplementary material available at 10.1186/s12913-022-08668-x.

## Background

Reducing child deaths is a global challenge [1]. Consequent to sequential measures taken to combat the causes of child deaths such as infectious diseases and malnutrition, the number of child deaths has been considerably reduced in high-income countries [2]. To further reduce child deaths, the extent to which the deaths are preventable has been investigated in some countries [3–6]. Examples of preventable deaths are drowning as a result of water activities without life jackets, a car accident without the appropriate use of a child seat, and a death despite neighbors’ frequent reports of child maltreatment. CDR is a system in which multidisciplinary agents work together to investigate, register, and review the deaths of all children, and suggest effective preventive strategies and interventions. Each participating agency delivers suggested preventive strategies and interventions to reduce similar preventable deaths. The agents typically involved are health facilities, law enforcement agencies, educational agencies, and child protection services. In Arizona, USA, a study conducted on the process of developing Child Death Review (CDR) from 1995 to 1999 reported that 29% of all deaths of children under 18 years of age could have been prevented, and preventability increased with the age of the child [7]. A pilot study on CDR in England, Wales, and Northern Ireland established that avoidable factors were present in 26% of all deaths of children under 18 years of age in 2006. Excluding neonates, the prevalence of potentially avoidable factors was as high as 43% [8]. Thus, there has been a focus on CDR as a method to reduce preventable deaths in children. CDR has been implemented in the USA, the UK, Australia, New Zealand, Canada, and other countries [3, 9–11]. Consequently, various preventive strategies have been recommended and executed, such as ensuring safety while sleeping to prevent sudden infant death syndrome and promoting traffic safety to prevent deaths due to traffic accidents [3]. A study in the USA reported that taking preventive actions based on CDR had reduced the deaths of children with whom child protective services had been involved [12]. The implementation of strategies suggested by CDR may further reduce the number of child deaths. However, various difficulties in applying CDR have been also identified, such as inadequate funding, time intensiveness, the need for committed personnel, difficulty in complete data collection, and lack of legislation [3, 10, 13].

In Japan, the number of child deaths decreased rapidly between the 1960s and 1980s, and the mortality rate of children below the age of five had become less than 10 per 1000 live births[14]. During this period child mortality had reduced due to the improvement in living and health service standards. Unintentional injuries, which are typical preventable deaths, are currently the major cause of deaths [15]. Various efforts have been made mainly by medical professionals to reduce preventable deaths from child abuse, accidents, and other external causes [16–18]. In this context, the interest in CDR has grown. In 2011, a study on the causes of child deaths was conducted across four regions of Japan, and 27.4% of deaths were identified as preventable [19]. The proportion of preventable deaths under the age of 18 was 25% in a 2014–2016 survey among pediatric specialty training facilities in Japan [20]. These results were similar to those reported by studies in the USA and UK [7, 8], while recommendations reflect the contextual issues of each country. In 2018, the Basic Law for Child and Maternal Health and Child Development was promulgated in Japan [21]. It provides measures for the collection of data and the maintenance of databases on child deaths. Accordingly, pilot CDR projects have been initiated across seven prefectures, starting in 2020 [22]. The core components of the CDRs remain the same with country where CDR had been previously introduced. The systems were tailored to the Japanese government system, wherein prefectures serve as the basic unit for key public services such as administration, health, education, police, and welfare services. However, the process of establishing the system faces various challenges. Through the pilot project, this study aimed to identify barriers and facilitators of the implementation of CDR system, and strategies to implement CDR more effectively in Japan.

## Methods

### Study setting: the pilot CDR project in Japan

Since April 2020, the pilot CDR project has begun in seven of the 47 prefectures in Japan, as a national project led by the Ministry of Health, Labour and Welfare [22]. Responding to an invitation by the national government, the seven prefectures voluntarily participated in the pilot project. The project’s budget is covered by the national government. The pilot CDR project in Japan is initiated by local governments, and the work is often outsourced to medical institutions or medical-related organizations. The CDR process consists of three stages: (1) data collection of child death cases, (2) review meetings, and (3) delivery of recommendations for preventive actions. The project aims at involving multiple agencies, such as child guidance centers, educational institutions, public health centers, and law enforcement agencies, in addition to medical experts such as pediatricians and forensic scientists. The participating agencies are supposed to carry out the recommended actions. Recommendations which fall on the agencies that do not participate in CDR are handled by the governor of local government. All deaths of children under 18 years of age are subject to a review in CDR. The CDR pilot project identifies all such deaths through notifications of the occurrence of child deaths provided by medical institutions and death records in national statistics [15, 23].

### Study design

This study uses the modified Delphi method to build consensus on barriers, facilitators, and implementation strategies of the nationwide CDR system initiation among experts in Japan. The Delphi method is typically used to synthesize the knowledge of experts when experimental methods to provide higher levels of evidence cannot be utilized [24]. In this study, the Delphi method was adopted since barriers, facilitators, and implementation strategies cannot be easily investigated by experimental studies given the nature of the public health policy of CDR. The modification made in this study was to start with a carefully-selected initial list of barriers, facilitators, and implementation strategies to minimize the burden of the expert panel. This modification is commonly utilized in health sciences [24–27].

### Study participants and data source

To recruit participants, we asked the local government officers responsible for CDR to select around five people who were deeply involved in the implementation and operation of the CDR in their respective prefecture. Hence, the inclusion criteria are: (1) involved in the CDR pilot project management or CDR review meetings, and (2) local government officers responsible for the CDR pilot project or those who are deeply involved in the pilot CDR project and selected by local government officers. Recruitment was carried out in October 2021.

A total of 39 experts were asked to participate in the Delphi expert panel, out of which 31 agreed. The 31 participants included 15 local government officers responsible for the pilot CDR project implementation and 16 health professionals responsible for the project in the organizations to which the CDR was outsourced. Health professionals comprised pediatricians, forensic scientists, emergency physicians, public health nurses, and medical social workers among others. At least one municipal representative and one project contractor representative from all pilot project prefectures participated.

This study was conducted from October to December 2021, one-and-a-half years after the initiation of the pilot CDR project, using the three-round modified Delphi method. In all the rounds, a survey was conducted using a web-based questionnaire. In each round, the invitation to the survey was sent to the entire expert panel who gave consent to participate regardless of their participation in the previous round. As a modification to the Delphi method, the lists of barriers, facilitators, and implementation strategies were prepared in advance based on an analysis of the seven project reports from the CDR pilot project, and semi-structured interviews with local government officers based on these reports. The project report is a structured report on CDR, submitted by the local government to the national government at the end of the fiscal year. The report is presented to the national government to identify issues that need to be addressed to scale up the CDR project throughout the country. The interviews were conducted by a researcher with a good understanding of the CDR project (HY). Based on the reports and transcription of the interviews, a content summarizing analysis was conducted by one researcher to extract barriers, facilitators, and implementation strategies to address them (HY). The extracted items were discussed amongst the researchers (HY, AA, KT) and the initial lists of barriers, facilitators, and combinations of barriers, facilitators, and implementation strategies to address the barriers or facilitators were developed. Implementation strategies were categorized by their implementation body— the national government and local governments. For example, the legislation for CDR was attributed to the national government, appointment of full-time staff to local governments, and gaining public awareness regarding both to the national and local governments.

The first round asked the panel to evaluate the importance of predefined lists of 13 barriers and 10 facilitators, and to collect additional barriers and facilitators. Importance was assessed using a 5-point Likert scale: (5) very important, (4) important, (3) neither important nor unimportant, (2) not important, and (1) not important at all. The index numbers demonstrate the scores of the items in terms of their importance. The barriers and facilitators to be added were then collected through open-ended questions. The specific questions from all rounds are presented in Table 1.

**Table 1:** Purposes and questions in each round

Purposes	Specific questions	Question types
Round 1		
Evaluation of barriers/facilitators included in the initial lists	We ask you about potential barriers/facilitators to CDR projects. When initiating and implementing a CDR project, how important do you think it is to take action on the following factors? [present the initial lists of barriers and facilitators]	5-point Likert scale range: Not important at all Not important Neither important nor unimportant Important Very important
Collection of additional barriers/facilitators	Please suggest any barriers/facilitators not included in the initial lists that may potentially obstruct or facilitate the CDR project.	Open-ended
Round 2		
Evaluation of barriers/facilitators suggested at round 1	We ask you about potential barriers/facilitators to CDR projects. When initiating and implementing a CDR project, how important do you think it is to take action on the following factors? [present the additional barriers and facilitators]	5-point Likert scale range: Not important at all Not important Neither important nor unimportant Important Very important
Collection of implementation strategies	We ask you about implementation strategies to address barriers/facilitators of CDR projects. Please suggest any other implementation strategies that you think would be effective to eliminate “barrier X”/ to realize “facilitator Y” other than the following strategies. [present the initial list of combinations of barriers/facilitators and implementation strategies to address them]	Open-ended
Round 3		
Evaluation of potential effectiveness of implementation strategies	We ask you about implementation strategies to address barriers/facilitators of CDR projects. How effective do you think the following implementation strategies are to eliminate “barrier X”/ to realize “facilitator Y”?	5-point Likert scale range: Not effective at all Not effective Neither effective nor ineffective Effective Very effective

The second round first asked the panel to ascertain the importance of additional barriers and facilitators. Importance was gauged using the same 5-point Likert scales from the first round. Subsequently, a total of 72 combinations of barriers, facilitators, and implementation strategies that were obtained in advance were presented. Implementation strategies were presented together with their implementation body (the national or local government). For example, when handling personal information in CDR act as a barrier against CDR implementation and legislation for CDR act as a potential implementation strategy to address the barrier, a combination of both factors, namely “difficulty in handling personal information in CDR (barrier)” and “legislation for CDR (implementation strategy)” is developed. As legislation is responsible for the national government, the combination is attributed to the national government. Implementation strategies to be added were collected from the panel. The combinations are presented in Tables 2 and 3.

**Table 2:** Barriers, facilitators and the national government-level implementation strategies and their priority

**Barriers**	**Implementation strategies**	**Importance score** **Mean**	**Effectiveness score** **Mean**	Priority score*
Lack of legislation for CDR	Legislation for CDR	4.90	4.96	0.11
	Constant budget allocations for CDR (Constant budget allocation implies that CDR is approved at a higher level)	4.90	4.79	0.24
	Official notices from the national governmental agencies that supervise local agencies involved in CDR	4.90	4.71	0.30
	Citizens’ acceptance for CDR (Citizen’s acceptance helps overcome difficulties caused by the absence of legislation)	4.90	4.54	0.47
Lack of legislation for collecting personal information for CDR	Legislation for CDR	4.90	4.96	0.11
	Development of personal information norms in addition to legislation for CDR	4.90	4.68	0.34
Inadequate collaboration of national governmental agencies involved in CDR	Legislation for CDR (Legislation formulates the involvement of multiple related agencies)	4.83	4.93	0.18
	National level multi-agency collaborations in CDR and sharing of CDR system development process	4.83	4.61	0.43
	Establishment of an integrated agency concerning children’s well-being and its reinforcement	4.83	4.54	0.49
Unstable budget allocations for CDR	Legislation for CDR (Legislation formulates budget allocation)	4.70	4.68	0.44
	Clarification of the amount and duration of CDR budget from the national government	4.70	4.57	0.52
Lack of monitoring and evaluation standards	Defining monitoring and evaluation indicators of CDR	4.52	4.32	0.83
	Development of guidelines regarding CDR output dissemination (Guidelines enable output dissemination, which is considered as achievement of CDR)	4.52	4.29	0.86
Lack of opportunities to educate child health professionals about preventable child deaths	Provision of education about preventable child deaths to professionals involved in CDR	4.52	4.46	0.72
Inadequate human and financial resources in the CDR program	Legislation for CDR (Legislation formulates human resource allocation)	4.50	4.61	0.64
	Provision of CDR forms, materials, and a data collection platform to local governments to minimize the workload	4.50	4.32	0.84
Involvement of families being undefined in CDR	Promotion of grief care methods to ensure appropriate contact with bereaved families	4.48	4.50	0.72
	Establishment of the way that CDR review results are delivered to the family	4.48	4.36	0.83
Lack of citizens’ acceptance	Legislation for CDR (Legislation becomes an opportunity to raise awareness)	4.47	4.86	0.55
	Raising public awareness using social and mass media	4.47	4.54	0.71
Reluctance toward multi-agency collaborations among agencies involved in CDR	Legislation for CDR (Legislation formulates the involvement of multiple agencies)	4.43	4.89	0.58
	Official notices from the national governmental agencies that supervise local agencies involved in CDR	4.43	4.54	0.73
Difficulty in collaboration between cities and prefectures regarding CDR	Legislation for CDR (Legislation overcomes ordinates specific to large cities)	4.30	4.89	0.71
	Official notices from the national governmental agencies that supervise local agencies involved in CDR	4.30	4.46	0.88
	Setting the appropriate-sized administration to implement CDR	4.30	4.32	0.97
Difficulty in understanding CDR personnel’s tasks and their workloads	Provision of training to local government officials and health professionals responsible for CDR	4.27	4.32	1.00
	Improvement in the quality of CDR materials to help understand the actual tasks (e.g., operation guidelines)	4.27	4.29	1.02
Implementation of preventive strategies recommended by CDR being not assured	Budget allocations to implement preventive measures recommended by CDR	4.24	4.71	0.81
	Creating a national database of past recommendations of preventive measures and implemented preventive measures to facilitate implementation of preventive measures at the national level	4.24	4.32	1.02
**Facilitators**	**Implementation strategies**	**Importance score Mean**	**Effectiveness ** **score ** **Mean**	Priority score*
Good multi-agency collaboration between agencies involved in CDR	Official notices from the national governmental agencies that supervise local agencies involved in CDR	4.67	4.64	0.49
CDR acceptance among professionals involved in CDR	Legislation for CDR (Legislation formulates the involvement of multiple agencies)	4.63	4.79	0.42
	Provision of training to local government officials and health professionals responsible for CDR	4.63	4.36	0.74
	Improvement in the quality of CDR materials such as operation guidelines	4.63	4.18	0.90
Top-down directions about CDR implementation	Legislation for CDR (Legislation urges higher level involvement)	4.27	4.89	0.74
	CDR outputs being embodied, visualized, and publicized to attract higher level attention	4.27	4.61	0.83
	Official notices from the national governmental agencies which supervise local agencies involved in CDR	4.27	4.50	0.89
Rewarding feelings in CDR-related work	The national government’s consistent positive attitude toward promoting CDR	4.27	4.68	0.80
Availability of support related to CDR	Continuous provision of support after the initial implementation phase	4.17	4.46	0.99
	Installation of a support team consisted of experts	4.17	4.43	1.01

*Priority scores were calculated using the distance between a coordinate (the mean importance score of a barrier or facilitator, the mean effectiveness score of an implementation strategy) and the coordinate (5,5). Smaller values indicate higher priority.

The barriers and facilitators which did not meet the consensus criteria were not shown in this table. Please see Supplementary Tables 1 and 2.

**Table 3:** Barriers, facilitators, and the local government-level implementation strategies and their priority

**Barriers**	**Implementation strategies**	**Importance score** **Mean**	**Effectiveness score** **Mean**	Priority score*
Lack of legislation for CDR	Constant budget allocations for CDR (Constant budget allocation implies that CDR is approved at a higher level)	4.90	4.57	0.44
	Citizens’ acceptance of CDR (Citizen’s acceptance helps overcome difficulties caused by the absence of legislation)	4.90	4.50	0.51
	Establish of embodied CDR operation guidelines by local governments to replace the law	4.90	4.43	0.58
Lack of legislation for collecting personal information for CDR	Obtainment of family consent to CDR (Family consent enables handling of personal information when there is no legislation for CDR)	4.90	4.32	0.69
Lack of opportunities to educate child health professionals about preventable child deaths	Provision of education about preventable child deaths to professionals involved in CDR	4.52	4.32	0.83
Inadequate human and financial resources in the CDR program	Appointment of a full-time staff to ensure workforce	4.50	4.61	0.64
	Provision of opportunities to exchange ideas about CDR to local government officials responsible for CDR so that CDR related tasks are efficiently managed	4.50	4.21	0.93
Involvement of families being undefined in CDR	Promotion of grief care methods to ensure appropriate contact with bereaved families	4.48	4.46	0.75
Lack of citizens’ acceptance	Raising public awareness using social and mass media	4.47	4.46	0.76
	Public awareness raising using posters, leaflets, and websites	4.47	4.14	1.01
Reluctance toward multi-agency collaborations among agencies involved in CDR	Higher-level agreements on CDR implementation among agencies involved in CDR	4.43	4.54	0.73
	Appointment of a full-time staff to ensure workforce to conduct multi-agency collaborations	4.43	4.43	0.80
	Reflecting opinions of agencies involved in CDR to develop recommendations of preventive measures	4.43	4.36	0.86
	Intensive efforts to explain and request cooperation with CDR to each agency	4.43	4.29	0.91
Difficulty in collaboration between cities and prefectures regarding CDR	Higher-level agreements on CDR implementation among agencies involved in CDR	4.30	4.32	0.97
	Appointment of focal people for CDR in particular local administrative divisions that are entitled to independent management	4.30	4.21	1.05
Implementation of preventive strategies recommended by CDR being not assured	Budget allocations to implement preventive measures recommended by CDR	4.24	4.64	0.84
	Establish monitoring systems about implementation of preventive measures recommended by CDR	4.24	4.07	1.20
**Facilitators**	**Implementation strategies**	**Importance score Mean**	**Effectiveness score Mean**	Priority score*
Good multi-agency collaboration between agencies involved in CDR	Appointment of a full-time staff to ensure workforce to conduct multi-agency collaborations	4.67	4.61	0.52
	Appointment of an appropriate unit in local governments for CDR (having multi-agency collaboration experiences)	4.67	4.39	0.69
	Intensive efforts to explain and request cooperation regarding CDR to each agency	4.67	4.39	0.69
	Efforts to exchange information frequently between agencies involved in CDR	4.67	4.29	0.79
CDR acceptance of professionals involved in CDR	Provision of information and training about CDR to professionals involved in CDR in the area	4.63	4.36	0.74
	Intensive efforts to explain and request cooperation regarding CDR to each agency	4.63	4.21	0.87
Pre-existing good multi-agency collaborations between agencies involved in CDR	Appointment of an appropriate unit in local governments for CDR (having multi-agency collaboration experiences)	4.50	4.32	0.84
	Select an organization with good multi-agency collaboration experience to outsource CDR	4.50	4.29	0.87
Leadership of local government officers	Appointment of a full-time staff to ensure workforce to take leadership	4.44	4.54	0.73
	Appointment of people who have appropriate skills and experiences as staff responsible for CDR	4.44	4.46	0.77
	Assurance that local government officials responsible for CDR can seek help and support from colleagues	4.44	4.46	0.77
	Provision of opportunities to exchange ideas about CDR to local government officials responsible for CDR	4.44	4.36	0.85
Top-down directions regarding CDR implementation	CDR outputs being embodied, visualized, and publicized to attract higher level attention	4.27	4.43	0.93
Rewarding feelings in CDR-related work	Realization of CDR results (e.g., implementation of recommended preventive measures, strengthened collaboration, etc.)	4.27	4.71	0.79
	CDR outputs being embodied, visualized, and publicized	4.27	4.54	0.87

*Priority scores were calculated using the distance between a coordinate (the mean importance score of a barrier or facilitator, the mean effectiveness score of an implementation strategy) and the coordinate (5,5). Smaller values indicate higher priority.

The barriers and facilitators which did not meet the consensus criteria were not shown in this table. Please see Supplementary Tables 1 and 2.

The final round asked them to evaluate the potential effectiveness of implementation strategies on the barriers and facilitators which they are meant to address. Hereafter, the effectiveness of implementation strategy refers to the potential effectiveness of implementation strategies on barriers or facilitators. Effectiveness was estimated using a 5-point Likert scale: (5) very effective, (4) effective, (3) neither effective nor ineffective, (2) not effective, (1) not effective at all. The index numbers show the scores of the items. To make sure that the effectiveness of implementation strategies was evaluated independently from their feasibility, the panel was instructed to assess effectiveness without considering feasibility. The implementation strategies themselves require various levels of effort to ensure the achievement of goals. Some of them are easy to realize, while others, such as legislation, require intensive efforts.

Additional barriers, facilitators, and implementation strategies, collected from the experts in the first and second rounds, were considered by three researchers regarding similarities and differences from existing barriers, facilitators, and implementation strategies (HY, AA, KT). When they were judged to be different from the pre-existing factors, they were evaluated in the next round.

### Data analysis

In this study, items were considered to reach consensus if the proportion of ratings 4 and 5 accounted for 85% or more for the barriers, facilitators, and implementation strategies. A consensus for combinations of barriers, facilitators and implementation strategies means that both barriers or facilitators and implementation strategies which consist of the combinations met the consensus criteria (proportion of rating 4 and 5 being above 85%). The Delphi methods typically use a cut-off value of 60% or more for consensus [24].

The average scores were calculated for the importance of barriers and facilitators, and the effectiveness of implementation strategies. For each combination of barriers, facilitators, and implementation strategies, the average value of their importance was plotted on the x-axis, the average value of the effectiveness of the implementation strategy was plotted on the y-axis, and the distance from the top-right point of the plotting field (coordinate (5, 5)) was calculated. The top-right point indicates a pair of a barrier or facilitator of the highest importance, and an implementation strategy of the highest effectiveness. Based on the distances, those with shorter distances were considered as higher priority combinations of a barrier or facilitator and an implementation strategy. For both the implementation bodies, the national and local government, higher priority combinations were identified.

## Results

### Summary of responses

The first round received responses from 30 out of 31 respondents (96.8% response rate), the second round received 25 out of 31 (80.6% response rate), and the final round received 28 out of 31 (90.3% response rate). The response rate was the lowest for the second round, which is probably a reflection of the volume of the combinations of barriers, facilitators, and implementation strategies in the questionnaire.

### Summary of the three rounds

**Fig. 1:**
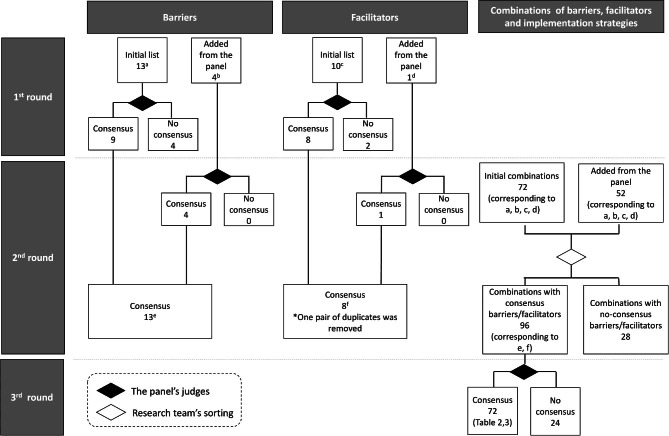
Consensus development flows of the barriers, facilitators, and combinations of barriers, facilitators, and implementation strategies. The complete list of barriers (a and b) is presented in Supplementary Table 1. The complete list of facilitators (c and d) is presented in Supplementary Table 2.

The first round presented 13 predefined barriers and 10 predefined facilitators to the panel, after which the panel evaluated their importance, of which, nine barriers and eight facilitators reached consensus. Upon the panel’s suggestions, four additional barriers and one additional facilitator were obtained. The second round presented additional barriers and facilitators, of which four additional barriers and one facilitator reached consensus. One facilitator was removed due to duplication. The second round also presented 72 combinations of barriers, facilitators, and implementation strategies, which are meant to address barriers or facilitators, and the panel suggested 52 additional implementation strategies. The third round asked the panel to evaluate the effectiveness of 96 implementation strategies on their corresponding barriers or facilitators, which reached consensus in the first and second rounds. Finally, 72 combinations reached consensus. The flows of consensus development are summarized in Figure 1. The complete lists of barriers and facilitators including those that failed to reach consensus are presented in Supplementary Tables 1 and 2

### Barriers

The 13 barriers are listed in Tables 2 and 3. Those that were determined to be of high importance to be addressed are as follows — “lack of legislation for CDR” (4.90 points), “lack of legislation for collecting personal information for CDR” (4.90 points), “inadequate collaboration of national governmental agencies involved in CDR” (4.83 points), and “unstable budget allocations for CDR” (4.70 points).

### Facilitators

The eight facilitators are listed in Tables 2 and 3, and those that were determined to be of high importance included “effective multi-agency collaboration between agencies involved in CDR” (4.67 points), “CDR acceptance among professionals involved in CDR” (4.63 points), and “effective pre-existing multi-agency collaborations between agencies involved in CDR” (4.50 points).

### Implementation strategies

The complete combinations are shown in Tables 2 and 3. The priority level was assessed using the importance and effectiveness scores. At the national government level, among barrier-strategy combinations, a combination of “lack of legislation” and “legislation for CDR” was at the highest priority (the importance score 4.90, the effectiveness score 4.96). Among facilitator-strategy combinations, a combination of “good multi-agency collaboration between agencies involved in CDR” and “official notices from the national governmental agencies that supervise local agencies involved in CDR” was at the highest priority (the importance score 4.67, the effectiveness score 4.64). At the local government level, among barrier-strategy combinations, a combination of “lack of legislation” and “constant budget allocations for CDR” was at the highest priority (the importance score 4.90, the effectiveness score 4.57), followed by a combination of “lack of legislation for CDR” and “citizens’ acceptance of CDR” (the importance score 4.90, the effectiveness score 4.50). Constant budget allocations imply that CDR is approved at a higher level, and citizens’ acceptance helps overcome the difficulties caused by the lack of legislation. Among facilitator-strategy combinations, a combination of “good multi-agency collaboration between agencies involved in CDR” and “appointment of a full-time staff” was at the highest priority (the importance score 4.67, the effectiveness score 4.61).

## Discussion

This study examined the barriers and facilitators of CDR implementation across pilot prefectures in Japan using a modified Delphi method along with the implementation strategies for these barriers and facilitators and reached expert consensus. It identified the importance of the lack of CDR legislation as a barrier, and acceptance among professionals and agencies involved in and establishing an effective collaborative relationship as facilitators. The study also found that legislative involvement is necessary for effective CDR implementation at the national government level. At the local government level, budget allocations, full-time staff, and citizens’ acceptance are important.

The study demonstrated that the legislation for CDR is the most important implementation strategy. The results reflect the opinion of experts that legislation is necessary for a wide variety of reasons. Legislation is expected to serve as the basis for the collection of personal information, the involvement of multiple agencies, the appointment of staff, and the allocation of a budget, among others. However, during the CDR pilot project, legislation referring to this detail was lacking. The Basic Law for Child and Maternal Health and Child Development, established in 2018 in Japan, is a conceptual law and does not establish specific requirements for CDR. Without specific legislation, local government officers and CDR contractors had to consider possible but less effective strategies to overcome the difficulty caused by the absence than legislation itself. This resulted in various implementation strategies paired with the barrier “lack of legislation.”

A few previous studies in other countries that have implemented CDR have emphasized on the importance of national legislation of CDR [3, 10, 28]. The International Society for the Prevention of Child Abuse & Neglect reported that CDR is practiced in 42 countries and regions around the world, but there are reportedly many areas that do not have CDR laws [5]. Where CDR laws exist, the matters required by law differ from country to country, and region to region [3, 5]. In the UK and USA, where CDR has been implemented ahead of other countries, in addition to the conceptual law, there are certain established guidelines that stipulate the delegation of authority to the team that actually performs CDR, or that have been specifically stipulated in CDR-related laws [11, 29]. To smoothly implement CDR, specific legislative action is needed, such as legislation for the collection of personal information.

To achieve the aims of CDR, the agencies involved must gather information to discuss and take preventive action. This study showed the importance of gaining acceptance among professionals and agencies involved in CDR, and establishing effective collaborative relationships. In the UK and USA, the engagement of motivated professionals and effective working relationships are said to be essential [5, 8, 11, 13, 29]. However, it is not easy to obtain the cooperation of multiple agencies due to issues surrounding the protection of personal information and a closed organizational culture. This study showed that useful measures to obtain cooperation from multiple agencies are leadership from local government officers who manage CDR, higher levels of agreement and commitment from them, and regular and smooth communication between child-related organizations. The importance of higher-level involvement was also reported by a previous study [28]. Thus, we need to build a more effective CDR while achieving better multi-agency collaboration.

For effective CDR implementation, it is important that citizens are well aware of it, including the bereaved families—this study indicated the importance of gaining the acceptance of citizens. This study also indicates the undefined involvement of bereaved families as being a barrier. For CDR, whether or not legislative action is taken, an explanation to the bereaved families and their subsequent cooperation are essential. Furthermore, some countries and regions try involving bereaved families in CDR’s review meetings to improve the quality of reviews using family perspectives [9]. However, they often face difficulties while attempting to involve families. The acceptance of CDR at a public level would reduce the negative responses on the part of bereaved families against CDR and facilitate the collection of personal information from multiple agencies. Involving citizens in CDR is also important, and specific positive impacts are noted as well. As an example, a few studies reveal that including citizen representatives in CDR would lead to initiatives such as recommending better preventive actions [3, 13]. Moreover, efforts to gain an understanding of the general community regarding CDR will result in cooperation toward conventional efforts to reduce child deaths, and preventive actions recommended by CDR. To achieve good citizen’s acceptance, it is necessary to consider public relations and other activities that will lead to public awareness and acceptance by the citizens.

As this study aimed to identify the barriers, facilitators, and their implementation strategies mainly during the establishment phase of CDR, it could not fully discern the factors that focus on running review meetings and suggesting recommendations, although some identified factors are equally important in the later stages. Implementation strategies of such kind in this study are constant budget allocations and the appointment of full-time staff. A previous study in the UK reported that different kinds of challenges emerge when CDR is operationalized, such as time intensiveness, clear remittance and purpose in each position, and the establishment of robust structures and processes [13]. Future examination of the processes involved in such a verification and the challenges faced when recommending preventive actions will provide a more complete overview.

Finally, the results of this study will be presented to both the national and local governments to accelerate CDR implementation. To help decision making, demonstrating the priority of combinations of barriers, facilitators, and implementation strategies is important. The CDR pilot project is supposed to be scaled-up in one year. The results will be utilized to support prefectures that will implement CDR in the next year.

To further reduce preventable child deaths, preventive actions developed through registration and review of such deaths are essential, especially in high-income countries. Understanding barriers, facilitators and implementation strategies of CDR will accelerate CDR implementation in Japan and guide CDR implementation in other countries, which is eventually expected to lead to decreased child deaths.

## Limitations

The Delphi method has some methodological weakness. Unlike experimental methodologies, it cannot infer causality. However, as CDR is a public health policy and experimental studies cannot be easily conducted, the Delphi method is the most reliable to reach a consensus on the importance of their barriers, facilitators, and the potential effectiveness of the implementation strategies. The modification we made also involved potential bias on the expert panel’s evaluations on barriers, facilitators, and implementation strategies that reached consensus as we developed and presented the initial lists. Although the initial lists were developed based on projects reports and interviews, the development was mainly conducted by a single researcher. However, the Delphi method ensured opportunities to reject the initially presented items and to add new items. Hence, the potential bias due to the modification and methodological weakness in preparation of the initial lists was minimized. As this study recruited the panel with the support of the local government officers responsible for CDR, there might have been selection bias. Those who became candidates might have had views which were closer to those of local government officers. However, to recruit a panel with profound experience on CDR, the support of the local government officers was crucial. The extrapolability of the results should be considered as the expert panel did not include personnel from all prefectures in Japan even though the panel was asked to evaluate barriers, facilitators, and implementation strategies for nationwide CDR implementation. For example, metropolitan cities were not equally included. Hence, context-specific issues might have been under-evaluated, which result in a failure to reach a consensus. Furthermore, the participating prefectures might have had a stronger interest in child mortality, which might have led to an underestimation of the barriers to CDR. Priorities were calculated based on the importance of barriers, facilitators, and the effectiveness of implementation strategies, but unequal patterns such as the weighting of importance and effectiveness was not considered. Some of the results of this study may also not be applicable to cultural settings in other countries. However, the major issues mentioned in the discussion, such as lack of legislation, multi-agency involvement, and citizens’ acceptance, were consistent with the results of previous studies conducted across different cultures.

## Conclusion

In this study, by combining the importance of barriers, facilitators, and the effectiveness of implementation strategies in the social implementation of CDR, high-priority implementation strategies were identified. To establish a framework for CDR, legislative action and effective handling of personal information are important as national government implementation strategies, and constant budget allocations, citizens’ acceptance, and the appointment of full-time staff are important as local government implementation strategies. This study demonstrated the importance of specific legislation for CDR, which serves as the basis of personal information handling, involvement of multiple agencies, appointment of staff, and allocation of budgets. The results also demonstrated various implementation strategies which are potentially effective in overcoming the difficulty caused by the absence of legislation. To better implement CDR, both calling for highly effective but difficult to realize strategies, such as legislation, and adapting less effective but feasible strategies need to be considered. CDR cannot be managed merely by individual responsibility or motivation; hence, it is important to establish a proper system to promote CDR in the future.

## Electronic supplementary material

Below is the link to the electronic supplementary material.

Supplementary Material 1

Supplementary Material 2

## Data Availability

The datasets generated and/or analyzed during this study are available from the corresponding author upon reasonable request.

## List of abbreviations

**CDR**: Child Death Review
**USA**: United States of America
**UK**: United Kingdom
