# Supplementary material for: Barriers, facilitators, and implementation strategies for the initiation of Child Death Review system in Japan: a modified Delphi method study
Source: BMC Health Serv Res. 2022 Dec 5;22:1482. doi: 10.1186/s12913-022-08668-x (PMC9724396; doi:10.1186/s12913-022-08668-x)
Supplement: Supplementary file 1 — Supplementary Material 1 [file 12913_2022_8668_MOESM1_ESM.docx]

**Supplementary Table 1. Complete list of barriers and their consensus**

| **Barrier** | **Consensus** |
| --- | --- |
| The initial lists presented to the expert panel in the first round |  |
| Lack of legislation for CDR | ○ |
| Lack of legislation for collecting personal information for CDR | ○ |
| Unstable budget allocations for CDR | ○ |
| Inadequate collaboration of national governmental agencies involved in CDR | ○ |
| Inadequate human and financial resources in the CDR program | ○ |
| Lack of citizens' acceptance | ○ |
| Reluctance toward multi-agency collaborations among agencies involved in CDR | ○ |
| Difficulty in understanding CDR personnel's tasks and their workloads | ○ |
| Difficulty in collaboration between cities and prefectures regarding CDR | ○ |
| Lack of detailed written procedures for operations | − |
| Difficulty in selecting feasible preventive measures | − |
| The difficulty and psychological burden of handling personal information | − |
| Difficulty in assembling for meetings because of locations of the agencies involved being far apart | − |
| Added from the expert panel in the first round |  |
| Lack of monitoring and evaluation standards | ○ |
| Lack of opportunities to educate child health professionals about preventable child deaths | ○ |
| Implementation of preventive strategies recommended by CDR being not assured | ○ |
| Involvement of families being undefined in CDR | ○ |
