# Supplementary material for: Barriers, facilitators, and implementation strategies for the initiation of Child Death Review system in Japan: a modified Delphi method study
Source: BMC Health Serv Res. 2022 Dec 5;22:1482. doi: 10.1186/s12913-022-08668-x (PMC9724396; doi:10.1186/s12913-022-08668-x)
Supplement: Supplementary file 2 — Supplementary Material 2 [file 12913_2022_8668_MOESM2_ESM.docx]

**Supplementary Table 2. Complete list of facilitators and their consensus**

| **Facilitator** | **Consensus** |
| --- | --- |
| The initial list presented to the expert panel in the first round |  |
| Good multi-agency collaboration between agencies involved in CDR* | ○ |
| CDR acceptance among professionals involved in CDR | ○ |
| Cooperation among a wide range of CDR-related organizations* | ○ |
| Pre-existing good multi-agency collaborations between agencies involved in CDR | ○ |
| Community members passionate about CDR before | ○ |
| Top-down directions about CDR implementation | ○ |
| Rewarding feelings in CDR-related work | ○ |
| Availability of support related to CDR | ○ |
| A high interest within the local community in the prevention of child deaths | − |
| Citizens' acceptance of CDR | − |
| Added from the expert panel in the first round |  |
| Leadership of local government officers | ○ |

***** “Good multi-agency collaboration between agencies involved in CDR” and “cooperation among a wide range of CDR-related organizations” were both contained in the initial list of facilitators, but later judged as being the same. Hence, “cooperation among a wide range of CDR-related organizations” was removed from the list of facilitators.
